# Transcriptome analysis of *Polianthes tuberosa* during floral scent formation

**DOI:** 10.1371/journal.pone.0199261

**Published:** 2018-09-05

**Authors:** Ronghui Fan, Yiquan Chen, Xiuxian Ye, Jianshe Wu, Bing Lin, Huaiqin Zhong

**Affiliations:** 1 Institute of Crop Sciences, Fujian Academy of Agricultural Science, Fuzhou, Fujian, China; 2 Flowers Research Center, Fujian Academy of Agricultural Science, Fuzhou, Fujian, China; 3 Fujian Engineering Research Center for Characteristic Floriculture, Fuzhou, Fujian, China; 4 Institute of Agricultural Engineering Technology, Fujian Academy of Agricultural Science, Fuzhou, Fujian, China; University of Naples Federico II, ITALY

## Abstract

*Polianthes tuberosa* is a popular ornamental plant. Its floral scent volatiles mainly consist of terpenes and benzenoids that emit a charming fragrance. However, our understanding of the molecular mechanism responsible for the floral scent of *P*. *tuberosa* is limited. Using transcriptome sequencing and *de novo* assembly, a total of 228,706,703 high-quality reads were obtained, which resulted in the identification of 96,906 unigenes (SRA Accession Number SRP126470, TSA Acc. No. GGEA00000000). Approximately 41.85% of the unigenes were functionally annotated using public databases. A total of 4,694 differentially expressed genes (DEGs)were discovered during flowering. Gas chromatography-mass spectrometry analysis revealed that the majority of the volatiles comprised benzenoids and small amounts of terpenes. Homology analysis identified 13 and 17 candidate genes associated with terpene and benzenoid biosynthesis, respectively. Among these, *PtTPS1*, *PtDAHPSs*, *PtPAL1*, and *PtBCMT2* might play important roles in regulating the formation of floral volatiles. The data generated by transcriptome sequencing provide a critical resource for exploring concrete characteristics as well as for supporting functional genomics studies. The results of the present study also lay the foundation for the elucidation of the molecular mechanism underlying the regulation of floral scents in monocots.

## Introduction

Floral scent is a crucial characteristic of ornamental plants, attracting pollinators for sexual reproduction and acting as a defense mechanism against pathogens, parasites, and herbivores [1–2]. Floral volatiles are mainly emitted by floral organs at specific flowering periods [3]. Approximately 1,700 floral volatiles from 100 plant species have been identified to date [1]. Floral volatiles consist of terpenoids, phenylpropanoids/benzenoids, and fattyacid derivatives, which are synthesized via different plant pathways [2].

Terpenes, the largest class of floral volatiles, are mainly composed of monoterpenes (C10), sesquiterpenes (C15), and diterpenes (C20)[3]. Volatile terpenoids are synthesized from two common isoprene precursors, namely, isopentenyl pyrophosphate (IPP) and dimethylallyl pyrophosphate (DMAPP). In the cytosol, IPP and DMAPP are derived from acetyl-CoA through the mevalonate (MVA) pathway and are catalyzed by farnesyl diphosphate synthase (FPPS) to form FPP. Then, cytosolic/mitochondrial terpene synthases (TPSs) convert FPP into various sesquiterpenes [4]. In plastids, IPP and DMAPP, which are synthesized from pyruvate and glyceraldehyde-3-phosphate by the 2-C-methyl-D-erythritol 4-phosphate (MEP) pathway, are converted into geranyl diphosphate (GPP) by GPP synthase (GPPS). Subsequently, plastidial TPSs catalyze the conversion of GPP into various monoterpenes and diterpenes [5]. The TPS gene family is further divided into seven subfamilies, namely, TPS-a to TPS-g [6–8]. Some TPSs involved in the biosynthesis of volatile terpenes have been investigated, including those in *Arabidopsis thaliana* (Arabidopsis) [9, 10], *Rose hybrida* (rose) [11], *Antirrhinum majus* (snapdragon) [12, 13], *Nicotiana suaveolens* (tobacco) [14], *Lilium spp* (lily) [15], and *Cananga odorata* (Ylang Ylang) [16].

Phenylpropanoids and benzenoids are the second largest class of floral volatiles that are generated in plastids via the shikimate pathway [17–18]. Phenylalanine ammonialyase (PAL) is the first committed step in the biosynthesis of benzenoids/phenylpropanoids and converts phenylalanine into *trans*-cinnamic acid [19]. The production of volatile benzenoids from cinnamic acid requires the shortening of the side chain by a C2 unit via the CoA-dependent β-oxidative pathway or CoA-independent non-β-oxidative pathway [19,20]. The β-oxidative pathway has recently been reported in the model plants *Petunia×hybrida* (petunia) [21–24] and *A*. *thaliana* (Arabidopsis)[25]. Current understanding of the non-β-oxidative pathway, including related enzymes, is limited.

*Polianthes tuberosa* is a member of the Agavoideae family that originated in Mexico and is mainly recognized for its aromatic oils and ornamental flowers [26]. Currently, it is cultivated in various tropical and subtropical regions. However, no genomic information on this species has been generated to date. When in bloom, the flowers of *P*. *tuberosa* emit an intense fragrance that consists of floral volatiles, mainly benzenoids such as methyl benzoate, methyl salicylate, methyl isoeugenol, and benzyl benzoate, and some terpenes, which include 1,8-cineol, farnesene, and germacrene D [27–30]. Previous studies have mainly focused on the composition analysis of floral scents, and the molecular mechanisms underlying the biosynthesis and regulation of these compounds remain unclear. To date, only two *PALs* and two *DXRs* have been identified in *P*. *tuberosa*[30]. Furthermore, the majority of research investigations that have focused on molecular mechanisms offloral scents involve dicots. In monocots, transcriptome analysis of floral scents has been reported for *Hedychium coronarium* (garland-flower)[31], *Cymbopogon flexuosus* (aromatic grasses)[32], *Vanda* Mimi Palmer (vandaceous orchid)[33], and *Lilium* ‘Siberia’ (lily)[34]. Thus, our understanding of the molecular mechanism underlying floral scent formation in *P*. *tuberosa* is limited.

*De novo* transcriptome analysis has become a useful tool in the discovery of genes involved in various metabolic pathways, including the determination of sequence and expression patterns without the need for a reference genome [35–37]. In the present study, we used RNA-seq technology to analyze the transcriptome of *P*. *tuberosa* flowers during four developmental stages. Four digital gene expression (DGE) libraries were obtained to analyze the gene expression patterns involved in floral scent formation during flowering. The results of this study can improve our understanding of the molecular mechanisms underlying floral scent formation in *P*. *tuberosa* as well as provide an important bioinformatics resource for investigating other biological mechanisms.

## Methods

### Plant material

**Fig 1:**
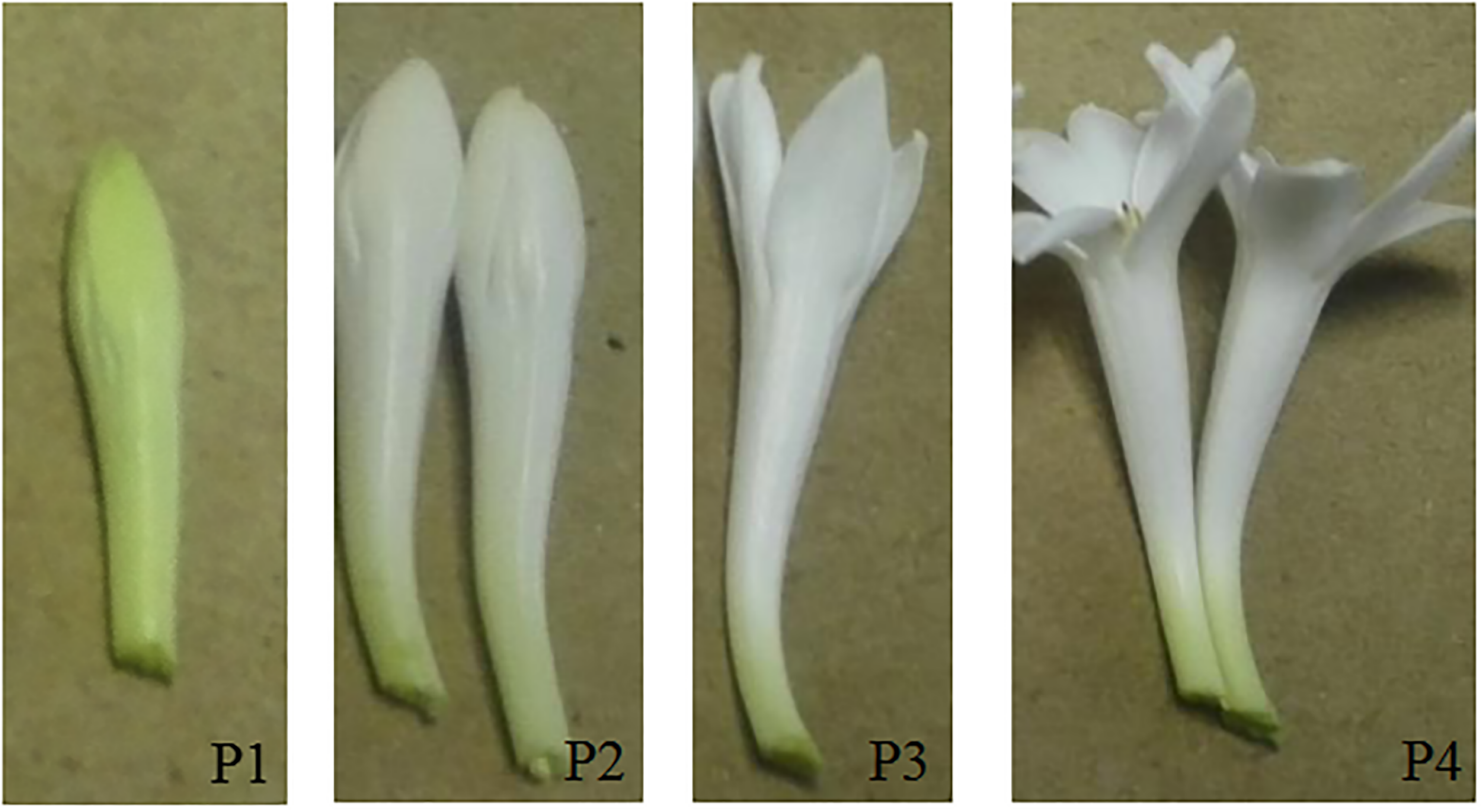
Floral developmental stages of *P*. *tuberosa*. P1, early bud stage; P2, mid-bud stage; P3, anthesis stage; P4, fully bloomed stage.

*P*. *tuberosa*, which was planted at the Fujian Academy of Agricultural Sciences (Fuzhou, China) in April, bloomed in August. The plant materials used in this study were collected on August 20, 2016 at 5:00 AM. The conditions of the sites included a daily high-low temperature of 35°C/29°C and a light-dark photoperiod cycle of 14 h/10 h. The materials were collected at four different flowering stages, immediately frozen in liquid nitrogen, and stored at -80°C. The four different flower stages were as follows: P1, early bud stage: completely closed petals, green; P2, mid-bud stage, completely closed petals, white; P3, anthesis stage: semi-open petals; and P4, fully bloomed stage: completely open petals (Fig 1).

### Headspace collection and gas chromatography-mass spectrometry (GC-MS) analysis

The volatile compounds were collected using a headspace-solid phase microextraction (HS-SPME)/GC-MS method. The whole flower was sealed in a 20-mL extraction bottle and equilibrated at 50°C for 10 min. The volatile compounds were extracted and adsorbed for 30 min using an SPME fiber [polydimethylsiloxane (PDMS), diameter 65 μm]. Then, the trapped floral scent compounds were analyzed using a Shimadzu GCMS-TQ8040 system. An Rxi-5Sil MS capillary column (30 m × 0.25 mm × 0.25 μm) was used for the separation of volatile compounds with helium as the carrier gas, at a flow rate of 1 mL/min. The column operating conditions were as follows: an initial 50°C hold step for 2 min, followed by an increase to 170°C at a rate of 4°C/min (held for 1 min), then to 270°C at a rate of 20°C/min, and a final hold step for 2 min. The volatiles were identified by comparing the mass spectra and retention times with authentic standards.

### RNA extraction, library construction, and RNA-seq

For transcriptome assembly, whole flowers of *P*. *tuberosa* (two biological replicates) were collected at the four flower developmental stages (S1 Fig). Total RNA was isolated using a universal RNA extraction kit (Bioteke Corporation, Beijing, China), and quality and quantity were examined with a NanoDrop 2000 UV-vis spectrophotometer (Thermo Scientific, MA, USA) and an Agilent 2100 Bioanalyzer (Agilent Technologies, CA, USA). Library construction was performed by the Biomarker Biotechnology Corporation (Beijing, China) using an mRNA-Seq sample prep kit (Illumina, CA, USA). Finally, the eight libraries were sequenced using an Illumina HiSeq^™^ 4000 platform.

### *De novo* assembly and functional annotation

Raw reads from the four samples were collected, and low-quality reads were removed. The remaining high-quality clean reads were then extended into longer contigs via their overlapping regions using Trinity software [38], further assembled into transcripts through pair-end joining, and then clustered to unigenes. Based on sequence similarity, all assembled unigenes were compared to public databases such as the NCBI non-redundant protein (Nr) and non-redundant nucleotide (Nt) databases, GO, eukaryotic orthologs groups (KOG), KEGG, clusters of orthologous groups of proteins (COG), Swiss-Prot protein database, protein family (Pfam), and orthologous groups of genes (eggNOG) using an E-value cut-off of 10^−5^.

### Expression annotation

The trimmed reads were aligned to the assembled transcriptome using Bowtie2 [39]. The abundance of each gene was estimated using RSEM (RNA-Seq by Expectation Maximization) [40]. Read counts per gene were calculated using fragments per kilobase of transcript per million mapped reads (FPKM) [41]. DEGs among eight different libraries were identified using DESeq [42]. An absolute false discovery rate < 0.01 and a fold-change value ≥ 2 were used as thresholds to confirm significant differences in expression levels.

### Q-PCR analysis

RT-qPCR was performed using the fluorescent dye SYBR Green (TaKaRa,China) on an ABI 7500 real-time PCR System (Applied Biosystems, USA). Primer sequences were designed using PrimerPremier 5.0 software. Actin-1 was employed as an internal control. The PCR conditions were as follows: 95°C for 30 s, followed by 40 cycles of 95°C for 5 s, 60°C for 34 s, 72°C for 30 s, and then 95°C for 15 s, 60°C for 1 min, and 95°C for 15 s. Each reaction was performed in triplicate. Relative expression levels were calculated using the 2^-ΔΔCt^ method [43].

## Results

### Identification of major floral compounds

To examine the composition of floral scents emitted by flowers, total volatile compounds from four flower developmental stages were analyzed by GC-MS. Low levels of benzenoids and terpenes were detected in green flower buds (P1), whereas these gradually increased at flower anthesis. At the anthesis stage (P3), the majority of the volatiles comprised benzenoids, coupled with lower amounts of terpenes such as methyl benzoate (17.07%), 1,8-cineole (10.00%), methyl salicylate (9.83%), germacrene D (7.68%), indole (6.65%), α-farnesene (4.92%), methyl anthranilate (4.29%), and methyl isoeugenol (3.56%) (Table 1).

**Table 1:** Relative abundance of volatile compounds in *P*. *tuberosa* flowers.

Compound	Retention time (min)	Relative amount (%) at specific flower developmental stages			
		P1	P2	P3	P4
Benzenoids					
Methyl benzoate	12.514	4.13 ± 0.20	26.75 ± 0.08	17.07 ± 0.21	7.49 ± 1.63
Methyl salicylate	16.292	3.28 ± 0.43	12.99 ± 0.23	9.83 ± 0.86	7.32 ± 0.32
Indole	19.959	—	1.03 ± 0.14	6.65 ± 0.02	9.78 ± 0.10
Methyl anthranilate	21.557	—	0.42 ± 0.01	4.29 ± 0.88	6.43 ± 0.42
Eugenol	21.949	—	0.28 ± 0.04	0.95 ± 0.01	1.13 ± 0.14
Trans-iseugenol	25.073	—	0.52 ± 0.04	2.91 ± 0.11	2.32 ± 0.36
Methyl isoeugenol	26.576	—	1.68 ± 0.06	3.56 ± 0.14	3.23 ± 0.08
Benzyl benzoate	34.181	0.35 ± 0.15	1.12 ± 0.11	1.09 ± 0.94	2.55 ± 0.10
Terpenes					
1,8-Cineol	9.948	—	4.46 ± 0.45	10.00 ± 0.39	9.42 ± 0.47
α-Terpineol	16.405	—	1.35 ± 0.28	1.91 ± 0.01	2.55 ± 1.17
Geraniol	18.457	—	0.28 ± 0.55	1.01 ± 0.08	1.15 ± 0.01
β-Bourbonene	23.032	0.47 ± 0.02	0.7 ± 0.08	1.38 ± 0.41	3.29 ± 0.80
Germacrene D	26.176	3.86 ± 0.26	5.85 ± 0.16	7.68 ± 1.41	5.75 ± 0.26
α-Farnesene	26.884	—	2.58 ± 0.11	4.92 ± 0.71	6.94 ± 0.10
Fatty acid derivatives					
(E)-2-Hexenal	2.81	7.53 ± 0.04	4.95 ± 0.11	3.51 ± 0.88	5.12 ± 0.13
2-Pentylfuran	8.273	6.24 ± 0.10	2.27 ± 0.23	1.34 ±0.21	0.99 ± 0.34
1,3-Hexadiene	9.869	13.03 ± 0.12	3.89 ± 0.30	—	—
(E)-2-Octenal	11.050	12.52 ± 0.40	4.87 ± 0.23	1.49 ± 0.48	2.44 ± 0.02
Nonanal	12.928	3.77 ± 0.45	1.99 ± 0.01	1.10 ± 0.12	1.54 ± 0.16
(E, Z)-2,6-Nonadiena	14.773	4.03 ± 0.49	2.76 ± 0.11	0.40 ± 0.03	1.81 ± 0.08
2-Nonenal	15.076	8.95 ± 0.41	3.12 ± 0.07	0.32 ± 0.04	1.73 ± 0.11
2,4-Decadienal	19.993	5.41 ± 0.32	4.03 ± 0.47	0.56 ± 0.02	0.35 ± 0.12

### *de novo* assembly, annotation of transcriptome, and identification of DEGs

Because no genomic information on *P*. *tuberosa* is currently available, transcriptome sequencing and *de novo* assembly were conducted in this study. A total of 228.71 million clean reads were generated from eight libraries (S4 Table). *De novo* assembly of these reads resulted in 96,906 unigenes with a mean length of 1,022 bp. The unigene details are shown in Table 2. Of the unigenes, 31,615 were longer than 1000 bp, 32.6% of the total.

**Table 2:** Quality parameters of the *P*. *tuberosa* transcriptome.

Length (bp)	Unigenes	
	Number	Percentage
200–300	9,519	9.82%
300–500	23,480	24.23%
500–1,000	32,292	33.32%
1,000–2,000	19,453	20.07%
2,000+	12,162	12.55%
Total number	96,906	
Total length	99,108,304	
N50 length	1,519	
Mean length	1,022.726	

Of the 96,906 unigenes, 40,563 assembled sequences were annotated using BLAST analysis against eight public databases, accounting for approximately 41.86% of all unigenes, with an E-value ≤1e-5. The annotation details are shown in S5 Table. In total, 23,396 unigenes were grouped into three major categories, molecular function, cellular components, and biological processes by GO annotation (S2 Fig). The number of annotated DEGs in P1 *vs*. P2 was higher than that in P2 *vs*. P3 and P3 *vs*. P4, suggesting that a large number of genes may be involved in flowering, particularly at the initial developmental stage. However, a large number of assembled sequences remained unannotated, which may be due to non-coding RNAs, untranslated regions, or specifically expressed in *P*. *tuberosa*. The special expression genes can be a great resource for the identification and study of novel genes [44, 45].

**Fig 2:**
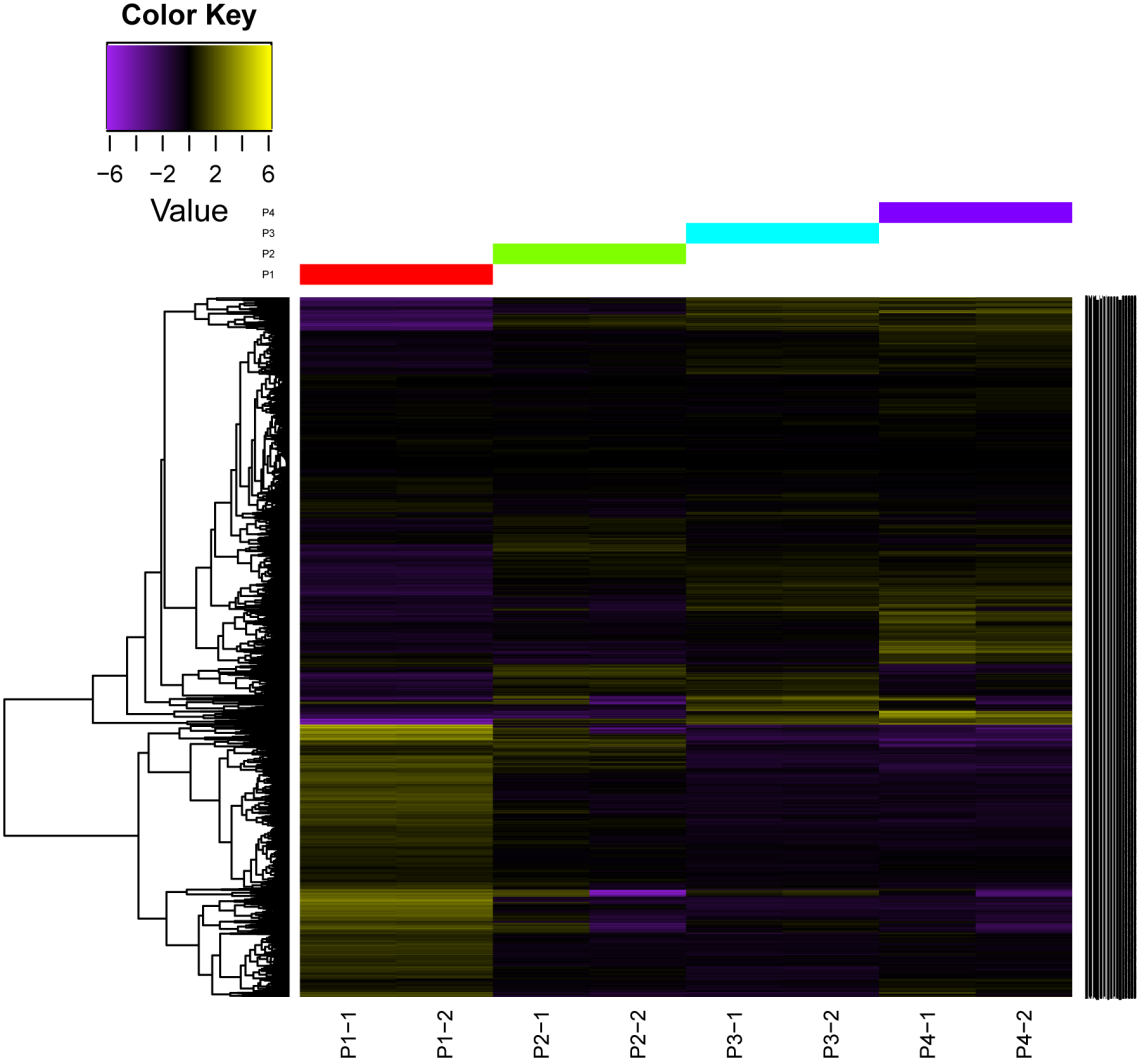
Expression profiles and cluster analysis of unigenes during four stages of flowering development.

Upon flowering, vast quantities of benzenoid volatiles accumulated in *P*. *tuberosa* from P1-P3, then decreased from P3-P4. To identify and select DEGs during flowering, the expression level of each gene in the eight libraries was compared in pairs of consecutive stages. The DEGs details are presented in S3 Fig. The number of DEGs in P1 *vs*. P2 was higher than that in P2 *vs*. P3 and P3 *vs*. P4, suggesting that more complex biological events occur during the initial stage of development. The transcript abundances were clustered by hierarchical cluster analysis (Fig 2). Expression analysis revealed that P1 had a pronounced difference from P2, P3 and P4, while a similar pattern was observed between P2, P3 and P4.

### Secondary metabolic pathways identified in flowering

**Fig 3:**
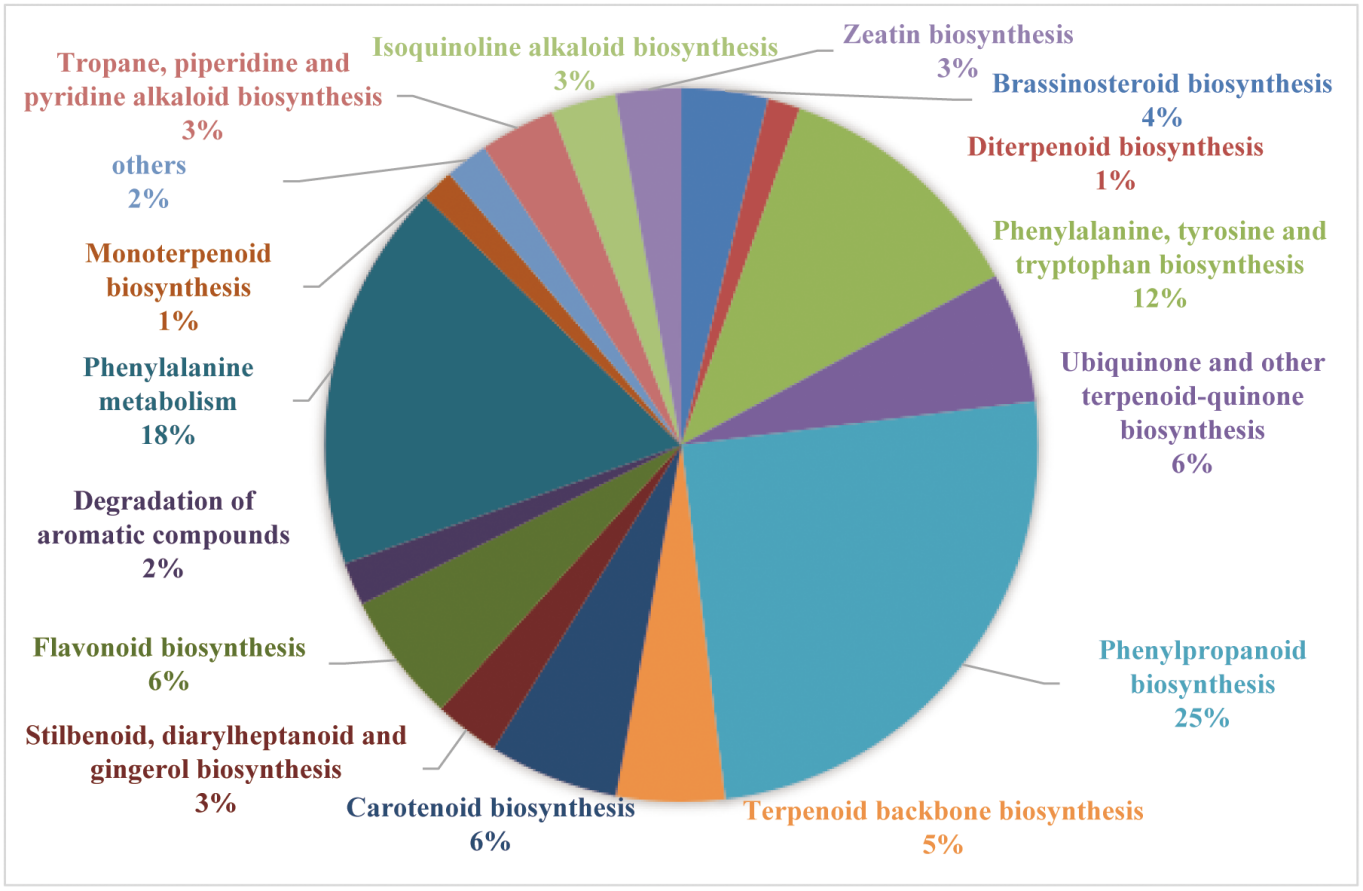
KEGG analysis of DEGs involved in secondary metabolism during flowering.

To identify unigenes related to secondary metabolic pathways, KEGG annotation was conducted by mapping the reference canonical pathways. About 14,771 (18.52%) unigenes were assigned to 113 KEGG pathways. Of these, 21 pathways of secondary metabolism were identified (184 DEGs). Phenylpropanoid biosynthesis and phenylalanine metabolism was the largest group (Fig 3), which was consistent with large amounts of benzenoid volatiles. Also, large numbers of DEGs involved in secondary metabolites, like terpenoid backbone biosynthesis, ubiquinone, and other types of terpenoid-quinone biosynthesis, monoterpenoid biosynthesis, and diterpenoid biosynthesis were represented, which was consistent with large amounts of some terpene volatiles. These unigene analyses are a valuable resource for gene mining and functional analysis of *P*. *tuberosa*.

### Identification of putative genes related to benzenoid biosynthesis

**Fig 4:**
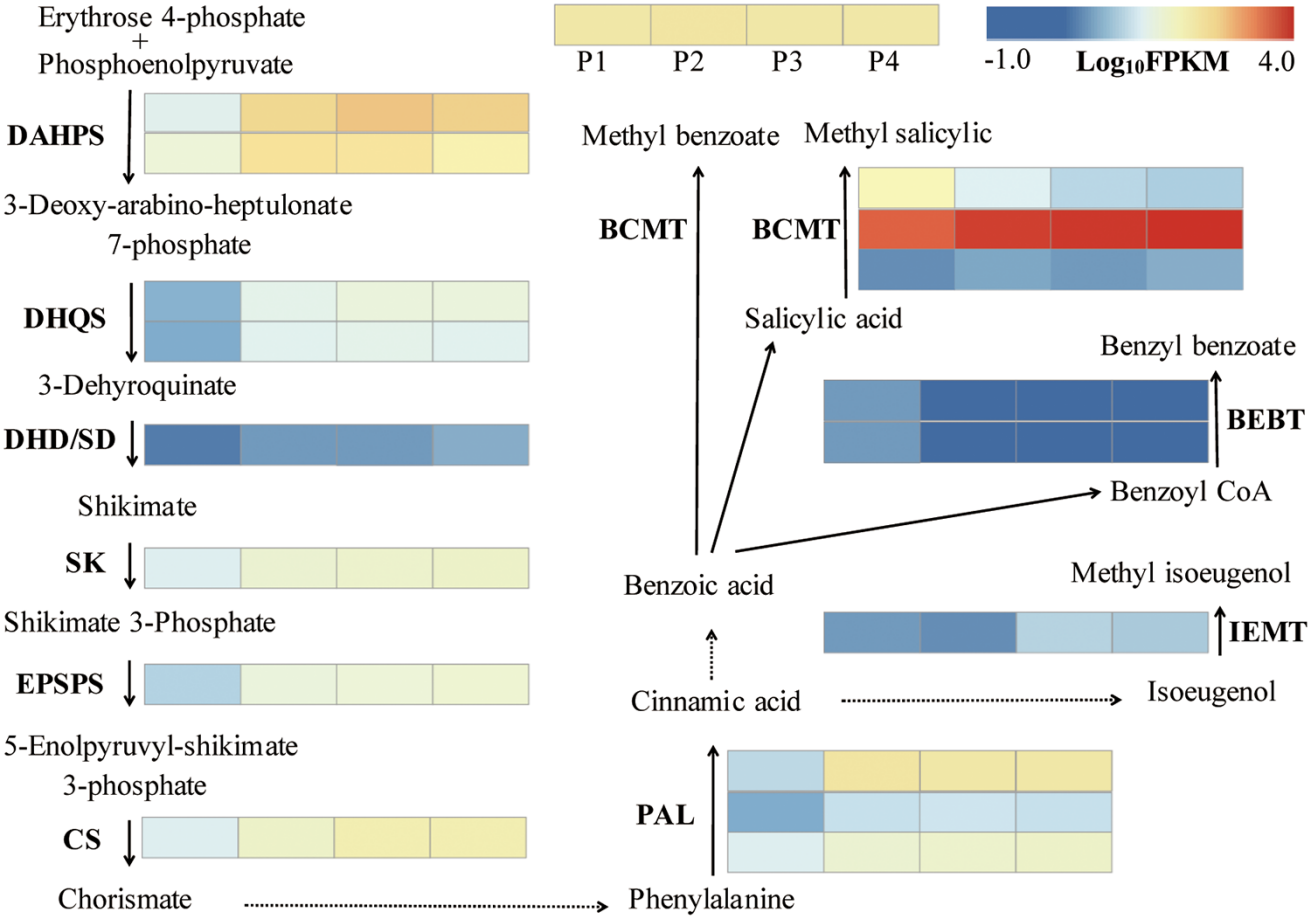
Expression profiles of genes in the shikimate and benzenoid biosynthesis.

Eight genes of the shikimate pathway, which supplies the carbon flux for benzenoid biosynthesis in plants, were here identified. These are two 3-deoxy-7-phosphoheptulonate synthases (*DAHPSs*) and 3-dehydroquinate synthases (*DHQSs*), one each of dehydroquinate dehydratase/shikimate dehydrogenase (*DHD/SHD*), shikimate kinase (*SK*), 3-phospho shikimate 1-carboxyvinyltransferase (*EPSPS*), and the chorismate synthase (*CS*) gene. The expression levels of the eight genes during flowering development are depicted in Fig 4. DAHPS represents the first reaction step, which converts erythrose 4-phosphate and phosphoenolpyruvate into 3-deoxy-arabino-heptulonate 7-phosphate [46]. Both PtDAHP1 and PtDAHP2 contained an N-terminal chloroplasttransit peptide (S4 Fig) and had a typical class-II DAHP synthase family domain (PLN02291). Homology analysis showed the predicted amino acid sequence of PtDAHP1 and PtDAHP2 to be very highly conserved in core sequences, and both of them were upregulated during flowering, suggesting that they play key roles in regulating the shikimate pathway. The following six genes were upregulated from the P1–P4 stages, indicating the predominant role of benzenoid volatiles in *P*. *tuberosa* (Fig 4, S5 Fig).

**Fig 5:**
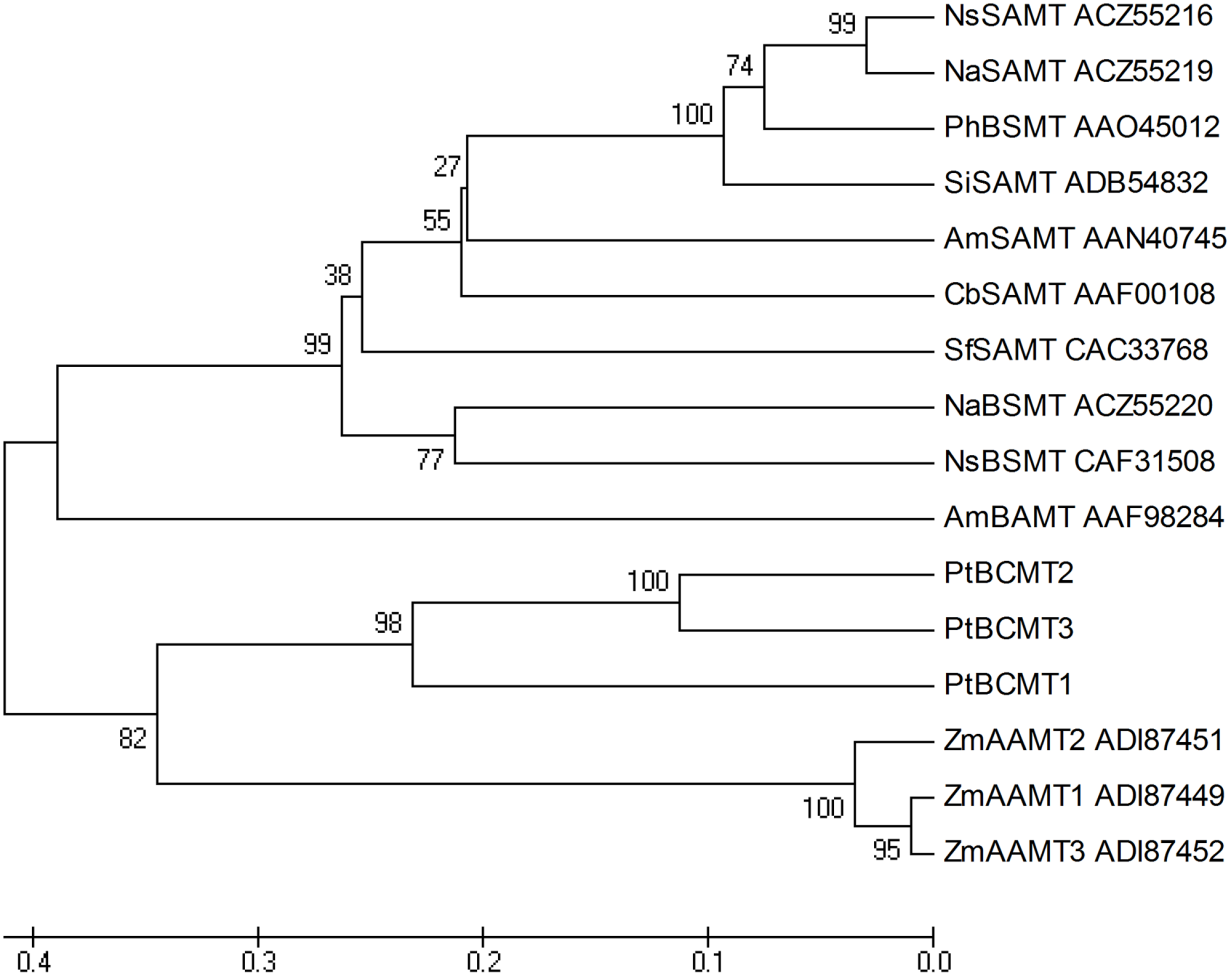
Phylogenetic reconstruction of the PtBCMTs and other BCMTs. Ns, *Nicotiana suaveolens*; Na, *N*.*alata*; Ph, *Petunia hybrida*; Sl, *Solanum lycopersium*; Am, *Antirrhinum majus*; Sb, *Sorghum bicolor*; Sf, *Stephanotis floribunda*; Zm, *Zea mays*.

To further investigate benzenoid biosynthesis in *P*. *tuberosa* and to confirm the identities of related genes in the pathway, the putative genes of benzenoid biosynthesis were investigated using sequence homology analysis. PAL is the first rate-limiting enzyme in benzenoid biosynthesis. It converts phenylalanine into cinnamic acid [19]. Three *PtPAL* genes were detected in the assembled transcriptome. Phylogenetic analysis of the corresponding amino acid sequences showed three PtPALs clustered into one phylogenetic group (S6 Fig), and their expression levels increased during flowering, particularly that of *PtPAL1*, which significantly increased from P1 to P2 and remained high through P3 and P4 (Fig 4). From the tree, PtPAL2 closely matched the PtPAL3. This may be due to gene duplication events, which may have mutated or diverged later from the common ancestor. In the final step, floral volatiles such as methyl isoeugenol, benzyl benzoate, methyl benzoate, methyl salicylate, and methyl anthranilate are synthesized. This process is catalyzed by S-adenosyl-L-methionine:(iso)eugenol O-methyltransferase(IEMT), benzyl alcohol benzoyl transferase (BEBT), and benzenoid carboxyl methyltransferase (BCMT). BCMT include salicylic acid methyltransferase (SAMT), benzoic acid carboxyl methyltransferase (BAMT), anthranilic acid methyltransferase (AAMT), and benzoic acid/salicylic acid carboxyl methyltransferases (BSMT) [47, 48]. Phylogenetic analysis of the deduced amino acid sequence showed that three PtBCMTs are very similar to maize AAMT but are different from other dicot BAMTs, BSMTs, and SAMTs (Fig 5). *PtBCMT2* was upregulated during the four stages of flower development, whereas the expression of *PtBCMT1* decreased and that of *PtBCMT3* remained low. The expression of two *PtBEBTs* decreased sharply during flowering development (Fig 4). *PtIEMT* was upregulated during flowering development, which coincides with the emission of methyl isoeugenol volatiles.

### Identification of putative genes related to terpene biosynthesis

**Fig 6:**
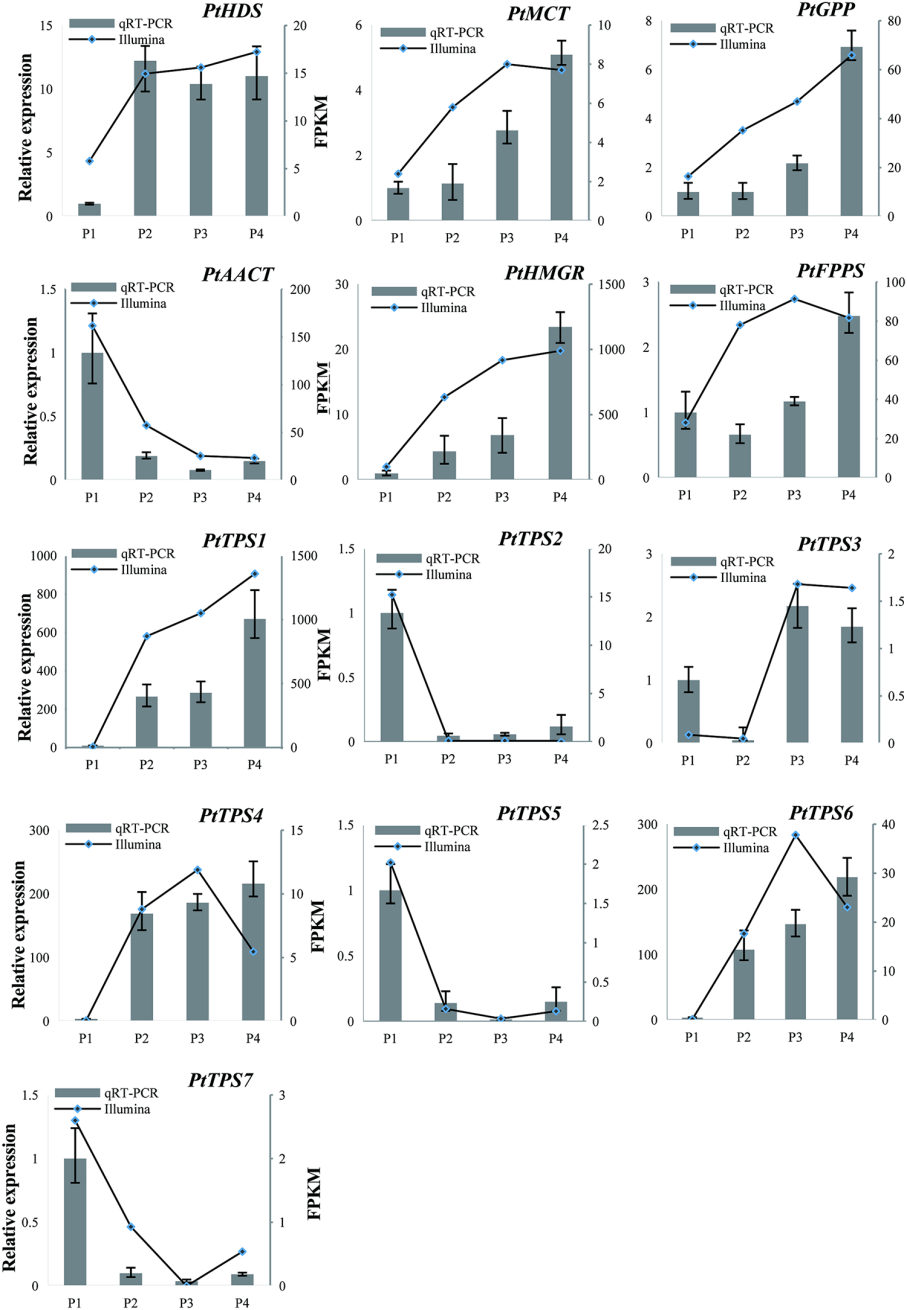
qRT-PCR validation of selected unigenes in the MEP, MVA, and terpene biosynthetic pathways.

The biosynthesis and emission of terpenes have been investigated in various plants. To establish further details on terpene biosynthesis in *P*. *tuberosa*, genes related to terpene biosynthesis were mined. The identified terpenoid biosynthesis genes involved in the MEP and MVA pathways were as follows: 4-hydroxy-3-methylbut-2-en-1-yl diphosphate synthase (*HDS*), *GPPS*, 2-C-methyl-D-erythritol 4-phosphate cytidylyltransferase (*MCT*), *FPPS*, acetyl-CoA acetyltransferase (*AACT*), and hydroxymethylglutaryl-CoA reductase (*HMGR*). DXS and AACT are the first enzymes of the MEP and MVA pathways, respectively. Six *PtDXS* were observed, all of which had very low expression levels, and *PtAACT* was significantly downregulated from P1–P4, indicating the minor role of terpene volatiles in *P*. *tuberosa*. PtGPPS and PtFPPS catalyze two common isoprene precursors (IPP and DMAPP) to produce GPP and FPP, which are the precursors of monoterpenes and sesquiterpenes [49]. Only one *PtGPPS* and *PtFPPS* was identified in the *P*. *tuberosa* transcriptome, which showed a 4.01- and 2.90-fold increase in expression from the P1 to P4 stages, respectively (Fig 6), indicating the crucial role in terpene biosynthesis.

**Fig 7:**
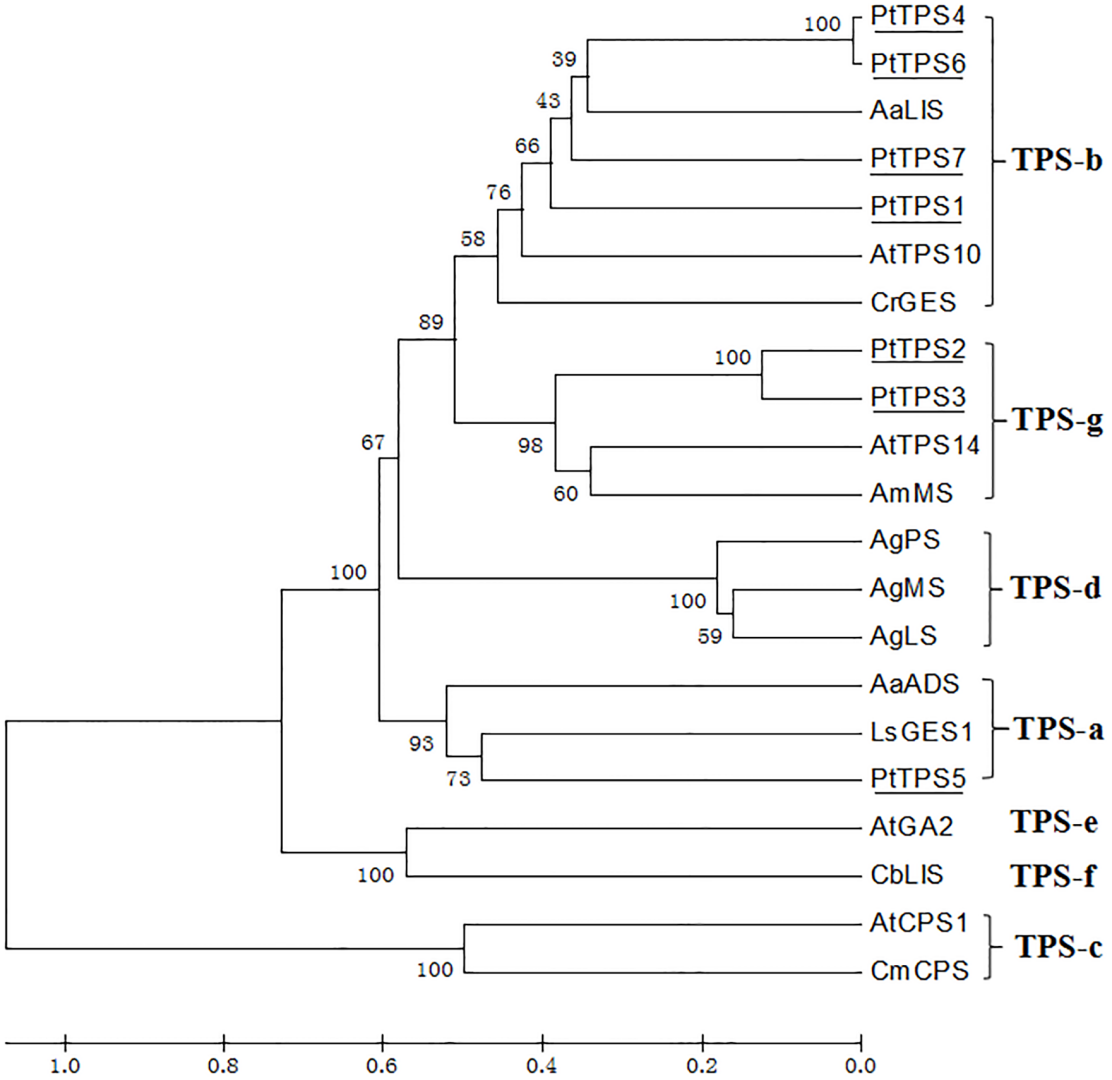
Phylogenetic analysis TPSs of *P*. *tuberosa*. A: Phylogenetic reconstruction of *P*. *tuberosa*TPSswith other plant TPSs using the MEGA 5 program; B: Alignment of deduced amino acid sequences of PtTPS2, PtTPS4, PtTPS5, and PtTPS6. AaLIS, linalool synthase (AAF13356); AtTPS10, *Arabidopsis thaliana* myrcene/ocimene synthase (AAG09310); CrGES, *Catharanthus roseus* geraniol synthase (AFD64744); AtTPS14, *A*. *thaliana* terpene synthase 14 (NP176361); AmMS, *Antirrhium majus* myrcene synthase (AAO41727); AgPS, *Abies grandis* pinene synthase (AAB71085); AgMS, *Abies grandis* myrcene synthase (AAB71084); AgLS, *Abies grandis* 4S-limonene synthase (AAB70907); AaADS, amorpha-4,11-dienesynthase (AFA34434); LsGES1, *Lactuca sativus* germacrene A synthase (AAM11626); AtGA2, *A*. *thaliana* ent-kaurene synthase (AAC39443); CbLIS, *Clarkia breweri S*-linalool synthase (AAC49395); AtCPS1, *A*. *thaliana* copalyl diphosphate synthase (NP_192187); and CmCPS, *Cucurbita maxima* copalyl diphosphate synthase (AAD04292).

TPSs exclusively catalyze GPP and FPP to monoterpenes and sesquiterpenes, respectively. *P*. *tuberosa* RNA-seq analysis identified four complete and three partial TPSs. Phylogenetic analysis of seven PtTPSs revealed that PtTPS1, PtTPS4, PtTPS6, and PtTPS7 could be clustered into the TPS-b subfamily, which consisted mainly of mono-TPSs, whereas PtTPS5 belonged to the TPS-a subfamily, which was composed of sesqui-TPSs, and PtTPS2 and PtTPS3 were assigned to the TPS-g subfamily (Fig 7). The deduced amino acids of PtTPS2, PtTPS4, PtTPS5, and PtTPS6 contained the motif DDXXD, which is a conserved domain in TPSs (S7 Fig). The expression of *PtTPS1* was significantly higher than that of the other six *PtTPSs*, suggesting that it played an important role in terpene biosynthesis.

**Fig 8:**
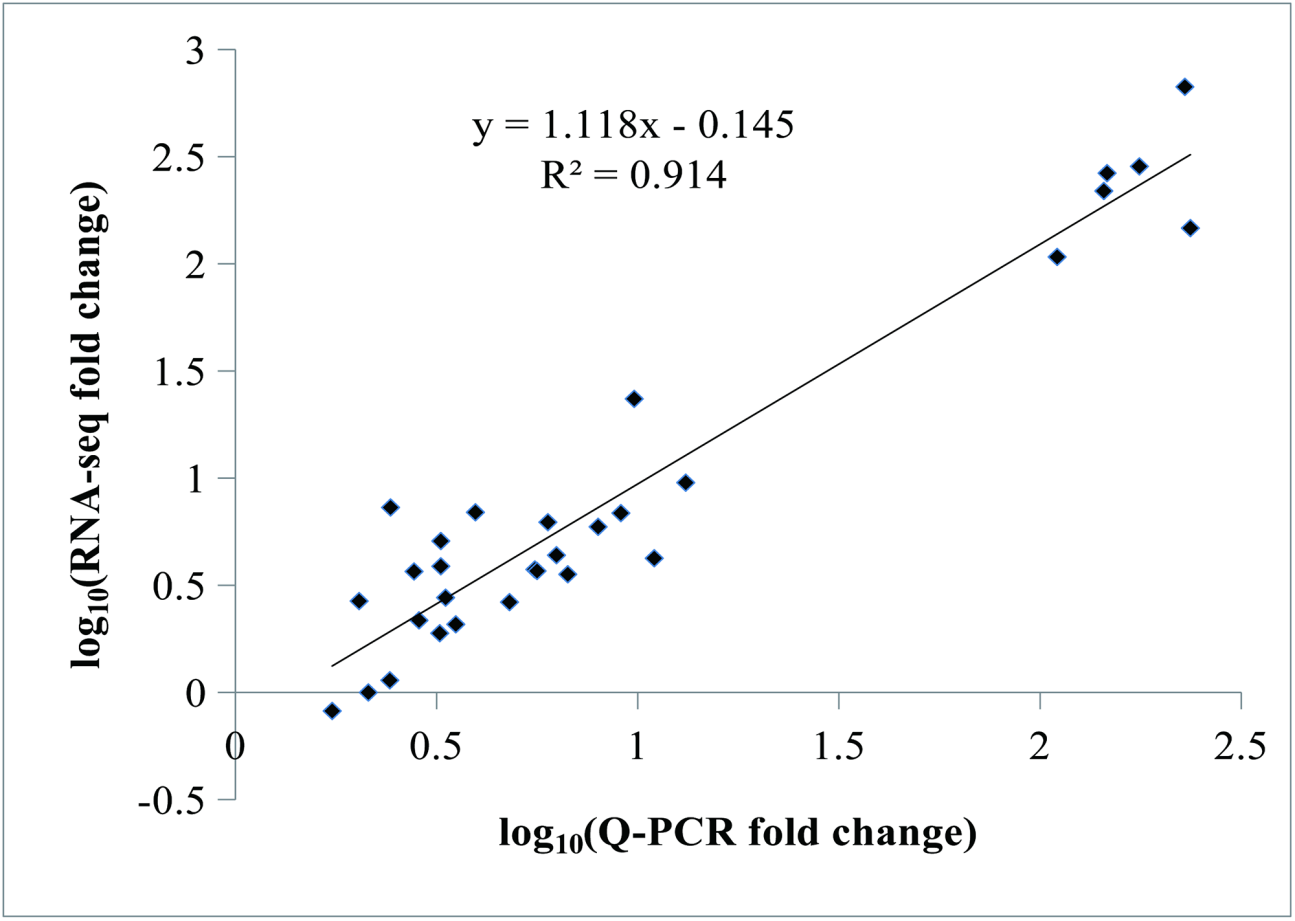
Scatter plot of 10 selected genes using RNA-seq and qRT-PCR analysis. The RNA-Seq fold-change represents the FPKM ratios of P2 (P3, P4) to P1; Q-PCR fold change refers to the relative expression from P2 (P3, P4) to P1.

Based on the log_10_ fold-changes in expression observable in the scatter plot (Fig 8), a positive correlation was observed between the results of qRT-PCR and RNA-Seq analyses (R^2^ = 0.914).

## Discussion

*P*. *tuberosa* is a popular flower with a distinct floral scent. However, our understanding of the molecular mechanisms responsible for its floral scent is limited because genomic information on this species is currently unavailable. The present study utilized extensive cDNA sequence data to identify genes that control floral scent compounds and to further analyze floral scent biosynthesis in *P*. *tuberosa*. Here, eight cDNA libraries during flowering of *P*. *tuberosa* were obtained through transcriptome sequencing. A total of 96,906 unigenes was obtained. Approximately 41.85% of the assembled unigene sequences were annotated by public databases. The availability of data for *P*. *tuberosa* provides an important resource for exploring the characteristics of this species as well as for mining key genes and their functions in floral scent development.

Floral scents mainly consist of terpenoids and benzenoids/phenylpropanoids, which are made up of a complex mixture of low-molecular-mass volatiles. The study of both volatile compounds and gene expression profiling have proven to be very powerful as a means of identifying candidate genes involved in the formation of floral scent. In *Arabidopsis*, Chen first detected floral volatile compounds from flowers. Then, candidate *AtTPS* genes were explored using the genomic information and RT-PCR [9]. In the absence of genome sequences, a combination of expressed sequence tags (EST) and metabolic profiling could be used to investigate scent-related genes, such as those in petunias [50], roses [11], and snapdragons [51]. In the present study, transcriptome sequencing combined with volatile compounds was used to explore floral scent formation. The results of GC-MS showed that the majority of floral volatile compounds in *P*. *tuberosa* were benzenoid compounds, which included methyl benzoate (17.07%), methyl salicylate (9.83%), indole (6.65%), methyl anthranilate (4.29%), and methyl isoeugenol (3.56%) (Table 1). High levels of some terpenes, including 1,8-cineole (10.00%), germacrene D (7.68%), and α-farnesene (4.92%), were also detected. This was consistent with the high volatile content previously reported in *P*. *tuberosa* flowers, which was attributed to benzenoid compounds and terpenes [29–30]. We also identified 13 and 17 candidate genes in *P*. *tuberosa* that were related to terpene and benzenoid biosynthesis, respectively. These genes and their expression patterns are an important resource for exploring floral scent in *P*. *tuberosa*. Several candidate genes, such as *PtTPS1*, *PtDAHPSs*, *PtPAL1*, and *PtBCMT2*, which are up-regulated and related to high contents of corresponding floral volatiles, might play crucial roles in floral scent.

The floral volatile compounds of *P*. *tuberosa* mainly include benzenoids/phenylpropanoids. In plants, the shikimate pathway, which provides metabolic flux to secondary metabolites, is the precursor in benzenoid biosynthesis. It is mostly regulated at the gene expression level [52]. DAHPS is the first enzyme in the shikimate pathway (Fig 4) [47]. It has been found that many plants contained two DAHPS genes, and the gene was upregulated in response to demands for increased emission of benzenoid floral volatiles [52–54]. In petunias, *PhDAHP1* was upregulated during flowering and RNA interference (RNAi) suppression of *PhDAHP1* revealed that emission of floral benzenoids decreased significantly, while RNAi of *PhDAHP2* in which transcript remained constitutive showed no change in emission of floral benzenoids, suggesting that *PhDAHP1* was responsible for the benzenoid/phenylpropanoid biosynthesis [54]. In *P*. *tuberosa*, there were a total of two DAHPS genes, *PtDAHPS1* and *PtDAHPS2*, which were significantly upregulated during flowering and remained at high levels in P3, appeared to play crucial roles in the shikimate pathway. The expression level of most genes in the shikimate pathway were upregulated during flowering. These were related to the high levels of benzenoid emissions, suggesting that the enzymes of shikimate pathway are important to the formation of benzenoid volatiles.

PAL catalyzed the transition of phenylalanine to trans-cinnamate and directed the carbon flow from the shikimate pathway to phenypropanoid metabolism. Several copies of PAL genes were found in some plant species, such as four genes in Arabidopsis, five in poplars and nine in rice [55]. The PAL genes had different functions in poplars and Arabidopsis, [56]. A total of three *PtPALs* were found in *P*. *tuberosa*, all of which had different expression patterns, consistent with their different functions. Of these, *PtPAL1* significantly increased from P1–P2 and remained high in P3 and P4, indicating the important role in the formation of benzenoid volatiles. BCMT, which catalyzes the biosynthesis of methyl benzoate, methyl salicylate, and methyl anthranilate, has been extensively investigated in petunia and snapdragon [31, 48, 49]. Phylogenetic analysis showed three PtBCMTs to be closely related to maize AAMT but distantly related to other BAMTs, BSMTs, and SAMTs (Fig 5). This may be because the enzymes in this group have broad substrate specificity and are more similar to each other in terms of catalyzing different substrates in this species than enzymes having the same functions in other species [57]. Only *PtBCMT2* showed sustained upregulation of expression. It also showed a positive correlation with the emission of methyl benzoate (Table 1, Fig 4). *PtBCMT2* seems to be primarily responsible for the biosynthesis of methyl benzoate. BEBT is the final enzyme in the biosynthesis of benzyl benzoate (Fig 4). The expression level of two *PtBEBTs* was low and decreased sharply during flowering, which coincides with the low benzyl benzoate content during flowering. Previous studies have shown the volatile emission in *P*. *tuberosa* to be rhythmic. Peak levels of benzyl benzoate were observed from evening until midnight, but these were relatively low during the day [30], and the expression level of BEBT was closely coordinated with benzyl benzoate emissions [30]. In our study, the activity of BEBT was low, which may reflect the time of our sample collection (5:00 AM).

DXS catalyzes the first step of the MEP pathway, which is the pathway upstream of monoterpene production. Transgenic plants accumulate different levels of monoterpene compounds than wild-type, so DXS acts as a limiting enzyme to control the biosynthesis of monoterpene [58]. In the present study, a total of six *PtDXS*s were identified, but all showed low expression levels. This may be attributable to the relatively low monoterpenecontents because benzenoid compounds are the main volatiles. TPSs convert GPP or FPP to various terpenes via a one-step method, and plants have a wide range of TPSs [59]. In *Hedychium coronarium*, most of the 17 *TPSs* were upregulated during flowering development [31]. We found approximately 18 TPSs from the transcriptome data of *P*. *tuberosa*. However, only 7 of the 18 PtTPS transcripts had relatively high expression levels, whereas the rest of the 11 exhibited very low expression levels. This may be because terpene volatiles are not the main volatile compounds of this species, and these genes have functions other than those involving volatile synthesis, and some have even completely lost their functions [59]. Some plant models possess 24–69 *TPS* genes [59]. Some TPS genes are expressed in specific tissues or need to be induced [6], indicating that there may be more TPS genes in *P*. *tuberosa* that have yet to be identified. TPSs are generally classified into seven clades, and TPSs mainly belong to only two or three clades in some plants, revealing the expansion of specific types of genes [59]. Among the 7 TPSs, four belong to TPS-b, one to TPS-a, and two to TPS-g. In higher plants, terpene emissions are regulated by the pattern of expression of TPS genes [4]. In snapdragons, the emission of volatile terpenes, such as (E)-β-ocimene and myrcene, are closely associated with expression of their related TPS genes during flowering [12]. *PtTPS1* which belonged to TPS-b (consisted mainly of mono-TPSs) showed the highest expression levels from P2 to P4, suggesting that this gene plays a crucial role in the biosynthesis of terpenes. The transcription level of *PtTPS1* showed positive correlation with the emission of 1,8-cineol, which is present in large quantities in terpene compounds (Table 1, Fig 6). It may be that PtTPS1s are cineol synthase, which are involved in1,8-cineol formation.

## Supporting information

S1 Table Primers used in qRT-PCR. (DOCX)

S2 Table Functional annotation of the unigenes. (XLS)

S3 Table Expression details of the unigenes. (XLS)

S4 Table Summary of Illumina transcriptome sequencing. (DOCX)

S5 Table Summary of unigene annotations. (DOCX)

S1 Fig Correlation heatmap of expression level in four stages of flowering. (DOCX)

S2 Fig GO enrichment analysis of DEGs during flowering. (DOCX)

S3 Fig DEGs in different stages of flowering. (DOCX)

S4 Fig Predicted amino acid sequence alignment of DAHPS. (DOCX)

S5 Fig qRT-PCR validation of selected unigenes in the shikimate pathway. (DOCX)

S6 Fig Phylogenetic analysis of PAL. (DOCX)

S7 Fig Predicted amino acid sequence alignment of TPS. (DOCX)

## Abbreviations

AACT: acetyl-CoA acetyltransferase
AAMT: anthranilic acid methyltransferase
BAMT: benzoic acid carboxyl methyltransferase
BCMT: benzenoid carboxyl methyltransferase
BEBT: benzyl alcohol benzoyl transferase
BSMT: benzoic acid/salicylic acid carboxyl methyltransferases
CS: chorismate synthase
DAHPS: 3-deoxy-7-phosphoheptulonate synthase
DHD/SHD: dehydroquinate dehydratase/shikimate dehydrogenase
DHQS: 3-dehydroquinate synthase
DMAPP: dimethylallyl pyrophosphate
EPSPS: 3-phospho shikimate 1-carboxyvinyltransferase
FPKM: fragments per kilobase of transcript per million mapped reads
FPPS: arnesyl pyrophosphate synthase
GPPS: geranyl pyrophosphate synthase
HDS: 4-hydroxy-3-methylbut-2-en-1-yl diphosphate synthase
HMGR: hydroxymethylglutaryl-CoA reductase
IEMT: S-adenosyl-L-methionine: (iso)eugenol O-methyltransferase
IPP: isopentenyl pyrophosphate
MCT: 2-C-methyl-D-erythritol 4-phosphate cytidylyltransferase
MEP: methyl-erythritol-phosphate
MVA: mevalonic acid
PAL: phenylalanine ammonialyase
SAMT: salicylic acid methyltransferase
SK: shikimate kinase
TPSs: terpene synthase
