# Supplementary material for: Transcriptome analysis of Polianthes tuberosa during floral scent formation
Source: PLoS One. 2018 Sep 5;13(9):e0199261. doi: 10.1371/journal.pone.0199261 (PMC6124719; doi:10.1371/journal.pone.0199261)
Supplement: S1 Table — (DOCX) [file pone.0199261.s001.docx]

| Gene ID | Gene name | Forward primer (5’-3’) | Reverse primer (5’-3’) |
| --- | --- | --- | --- |
| Unigene_19761 | PtGPP | AGATTGAAAGGCTGAGGCGGTAC | AGCTGCTCCTTGGCGTCCCT |
| Unigene_85922 | PtHDS | CGGCAGTGAGCACCCGATAA | TGAATGTCCGCCACCAGAGG |
| Unigene_91994 | PtMCT | CCCACTATTAGGACAACCAA | AAGTTCCGAATATCCATCAA |
| Unigene_83731 | PtFPP | TACCTGGAGGAAAGCTAAAC | CTTGGAGCCATTCGATACAC |
| Unigene_26600 | PtAACT | GGTGCTGGAATACCAAATAC | ATCCCATCAACAAGAGTGTC |
| Unigene_06634 | PtHMGR | AAGTCAGTGGTATGTGAGGCAATC | GCCAGTGGCAATGAAGACAG |
| Unigene_13233 | PtTPS1 | TCAGGGATGTATGATGTGCT | ATGGGAATTTACAAGTCCTTTC |
| Unigene_68642 | PtTPS2 | TTTTGGGTGAAGGGATTACG | TTTGAGCAGAGTGCTGTGGG |
| Unigene_84362 | PtTPS3 | AATTCTTCGGCTTTGGGATG | CAAGATTGGGAGCGAAATGA |
| Unigene_16999 | PtTPS4 | CACTCGTCTATGCTCCTTCG | AACCATCTCGCTTATCTGCT |
| Unigene_13044 | PtTPS5 | ACTTTGTCTCCCTTCGGTTTC | AGTTTGCAGCGTCGTCTAAT |
| Unigene_39728 | PtTPS6 | CACTCGTCTATGCTCCTTCG | TCTCGCTTATCTGCTCACAA |
| Unigene_63066 | PtTPS7 | GGCTTATGCCATGATGCTAA | TGAGATGCCTGACACTGAAT |
| Unigene_02713 | PtDAHPS1 | AAGAAGGGAGCCACCCAGGAG | CGGGAGCAAAGGTCGTCAAA |
| Unigene_34333 | PtDAHPS2 | AGGATCTGGAGACGGTGCTG | TGAAGGTGTCCCTGATGTTGTT |
| Unigene_10130 | PtSK | ATGATGGAGGCTACTGCTGC | GATGAATGGAAGTGCGGTGA |
| Unigene_31918 | PtDHQS1 | GGATTTGGCTATGGGCAGTG | TCTCAGGTGGAGCAGTTGGA |
| Unigene_37185 | PtDHQS2 | AGGAAAGTGGAGTGAGGGCTAC | TGCTGCAAGATTCGGAGAAC |
| Unigene_37871 | PtEPSPS | TGGCTGTCGTTGCTCTGTAT | TGTTGCTCCCAGCTTTCTTA |
| Unigene_40672 | PtDHD/SHD | CCAAACTGGGAAGGAGGTCA | TGCGTGCTACATCACAAATGTCTA |
| Unigene_35104 | PtCS | TGCCCTCATCCTTCTCGTCC | TGGCCTGGCCTCCTTCTATC |
| Unigene_52528 | PtBEBT1 | CTCCCAAAGTGCATCCTG | CTTCAAAGTGGCGATGTCT |
| Unigene_90522 | PtBEBT2 | TGCTGGCTACTACGGTAATGC | CCAGTGGTATCCGAAACAAAG |
| Unigene_23691 | PtBCMT1 | GAAACAAGTTATGCCACCAA | AGCAGTAGGACCAGATGAGC |
| Unigene_32872 | PtBCMT2 | GCCGATCTGGGTTGTTCATC | CATTTCCAGGGAGGTCGTTC |
| Unigene_62522 | PtBCMT3 | AGAGGAGGAAAGAGTGGATG | CCCAGTTCAACTCGAAAGTA |
| Unigene_14565 | PtPAL1 | CCAACATTCTCGCCGTCCTA | GGGTGGTGCTTCAGCTTGTG |
| Unigene_88002 | PtPAL2 | CCGACGGCAAGGCTATCAAC | ACCCCACGGCGGTGCCGTTGA |
| Unigene_35211 | PtPAL3 | CCGACGGCAAGGCGATCGAT | ACCCCACGGCGGTGCCGTTCA |
| Unigene_68795 | PtIEMT | TATTTGGAGACCTTAGCA | TTACGCAGCTCTATCACT |
| - | Actin-1 | CTATGAACTGCCTGATGGAC | TGGAGTTGTAGGTTGTTTCG |

Table S1 sequences of primers used in this experiment
