# Supplementary material for: Transcriptome analysis of Polianthes tuberosa during floral scent formation
Source: PLoS One. 2018 Sep 5;13(9):e0199261. doi: 10.1371/journal.pone.0199261 (PMC6124719; doi:10.1371/journal.pone.0199261)
Supplement: S4 Table — (DOCX) [file pone.0199261.s004.docx]

| **Sample** | **Total Bases** | **Clean Reads** | **GC content** | **% ≥Q30** |
| --- | --- | --- | --- | --- |
| **P1-1** | 7,944,054,912 | 26,747,781 | 48.49% | 90.80% |
| **P1-2** | 9,176,911,674 | 30,775,042 | 48.10% | 89.95% |
| **P2-1** | 8,112,305,270 | 27,282,315 | 48.69% | 90.58% |
| **P2-2** | 7,429,491,452 | 25,041,991 | 48.55% | 91.10% |
| **P3-1** | 8,655,760,330 | 29,115,723 | 49.11% | 90.47% |
| **P3-2** | 8,081,365,754 | 27,246,139 | 48.95% | 91.07% |
| **P4-1** | 10,019,931,304 | 33,622,255 | 48.14% | 90.68% |
| **P4-2** | 8,588,274,262 | 28,875,457 | 48.23% | 91.13% |
