# Supplementary material for: Transcriptome analysis of Polianthes tuberosa during floral scent formation
Source: PLoS One. 2018 Sep 5;13(9):e0199261. doi: 10.1371/journal.pone.0199261 (PMC6124719; doi:10.1371/journal.pone.0199261)
Supplement: S5 Table — (DOCX) [file pone.0199261.s005.docx]

| **Database** | **Number of annotated unigenes** | **Percent of annotated unigenes (%)** |
| --- | --- | --- |
| **COG** | 12,290 | 15.41 |
| **GO** | 23,396 | 29.33 |
| **KEGG** | 14,771 | 18.52 |
| **KOG** | 22,590 | 28.32 |
| **Pfam** | 27,076 | 33.94 |
| **Swiss-Prot** | 25,712 | 32.23 |
| **eggNOG** | 37,360 | 46.83 |
| **Nr** | 39,943 | 50.07 |
| **All** | 40,563 | 50.85 |
