# Supplementary figures and images for: Transcriptome analysis of Polianthes tuberosa during floral scent formation
Source: PLoS One. 2018 Sep 5;13(9):e0199261. doi: 10.1371/journal.pone.0199261 (PMC6124719; doi:10.1371/journal.pone.0199261)

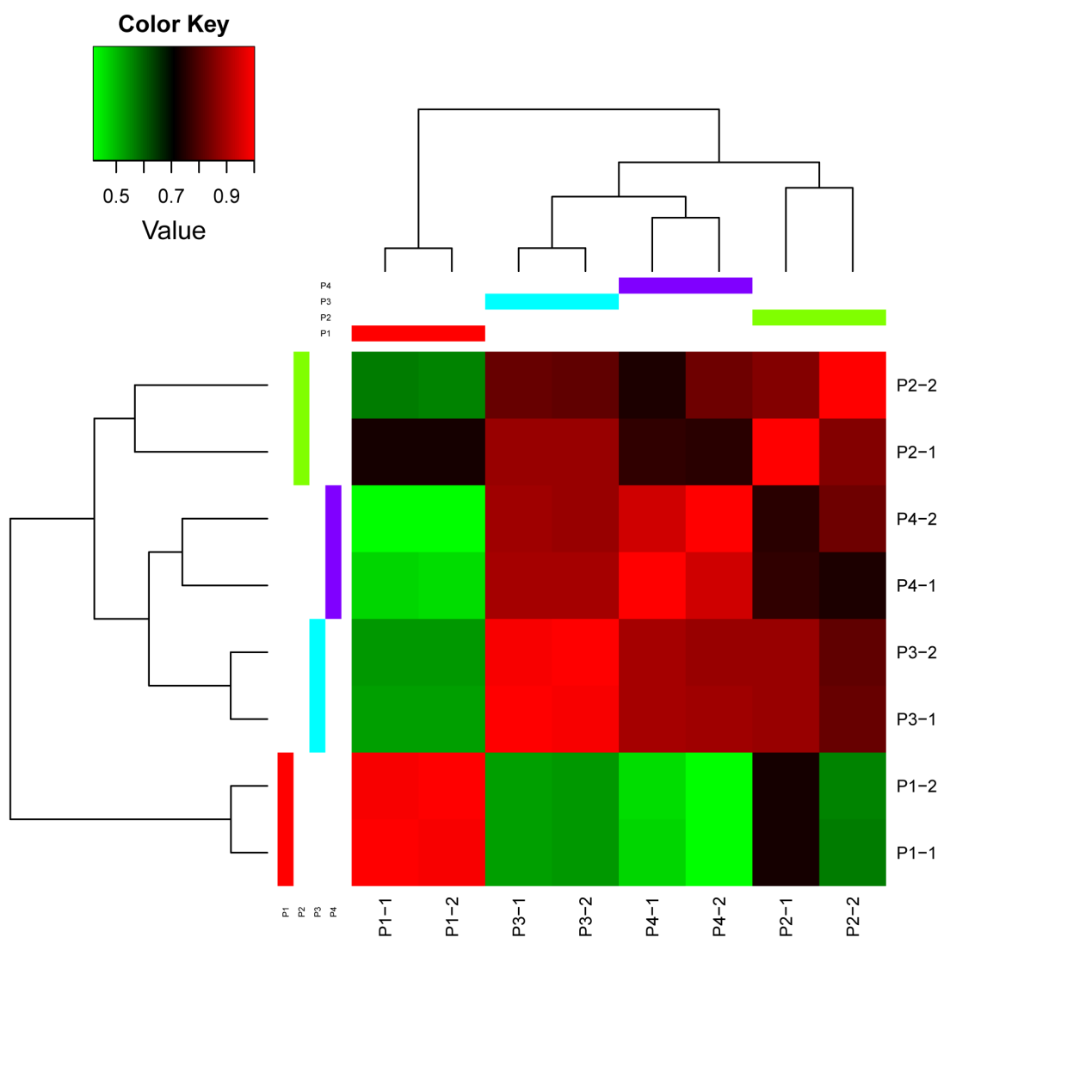

Supplement: S1 Fig — (DOCX) [file pone.0199261.s006.docx]

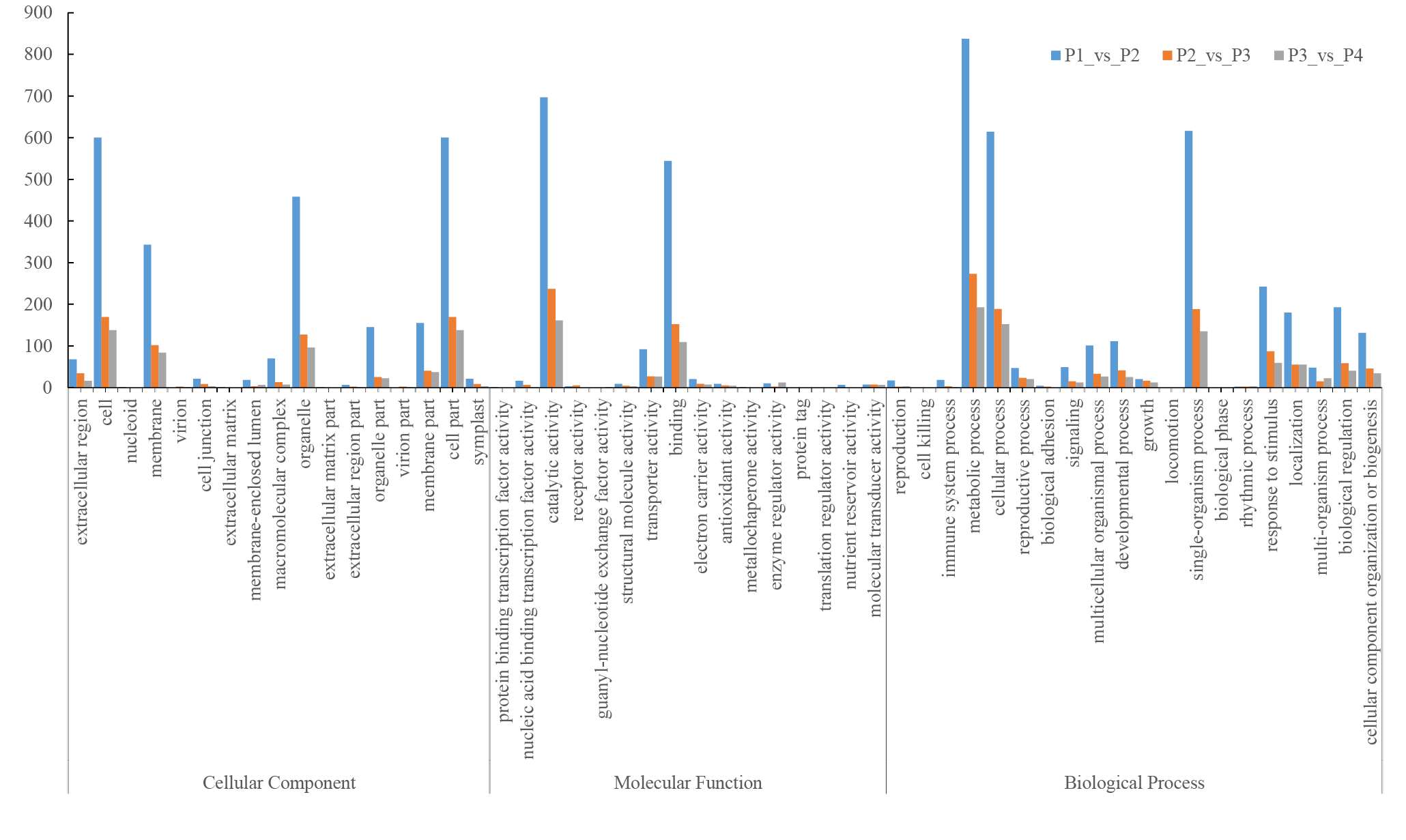

Supplement: S2 Fig — (DOCX) [file pone.0199261.s007.docx]
