# Supplementary material for: Transcriptome analysis of Polianthes tuberosa during floral scent formation
Source: PLoS One. 2018 Sep 5;13(9):e0199261. doi: 10.1371/journal.pone.0199261 (PMC6124719; doi:10.1371/journal.pone.0199261)
Supplement: S3 Fig — (DOCX) [file pone.0199261.s008.docx]

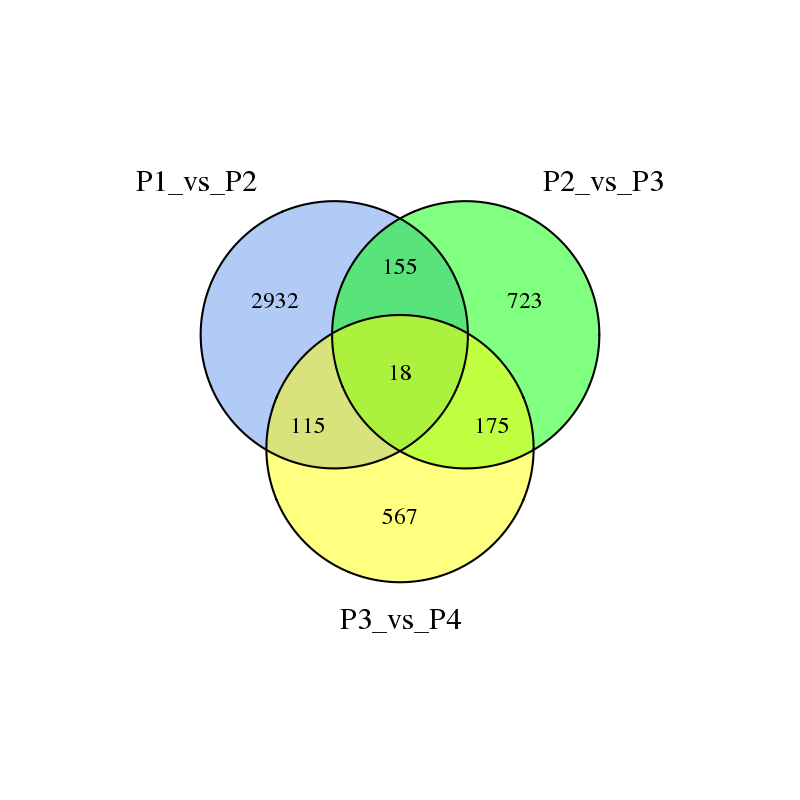


**A**

**B**

1. Venn diagram of DEGs at different stages of flower development. (B) The expression patterns of DEGs at different stages of flower development
