# Supplementary material for: Transcriptome analysis of Polianthes tuberosa during floral scent formation
Source: PLoS One. 2018 Sep 5;13(9):e0199261. doi: 10.1371/journal.pone.0199261 (PMC6124719; doi:10.1371/journal.pone.0199261)
Supplement: S4 Fig — (DOCX) [file pone.0199261.s009.docx]

# AtDAHP1: *Arabidopsis thaliana*, AEE87148; AtDAHP2: *Arabidopsis thaliana*, AEE86237;

NtDAHP: *Nicotiana tabacum*, NP_001312132; PhDAHP1: *Petunia x hybrida*, AFL02467 ; PhDAHP2: *Petunia x hybrida*, AFL02468.
