# Supplementary material for: Transcriptome analysis of Polianthes tuberosa during floral scent formation
Source: PLoS One. 2018 Sep 5;13(9):e0199261. doi: 10.1371/journal.pone.0199261 (PMC6124719; doi:10.1371/journal.pone.0199261)
Supplement: S6 Fig — (DOCX) [file pone.0199261.s011.docx]

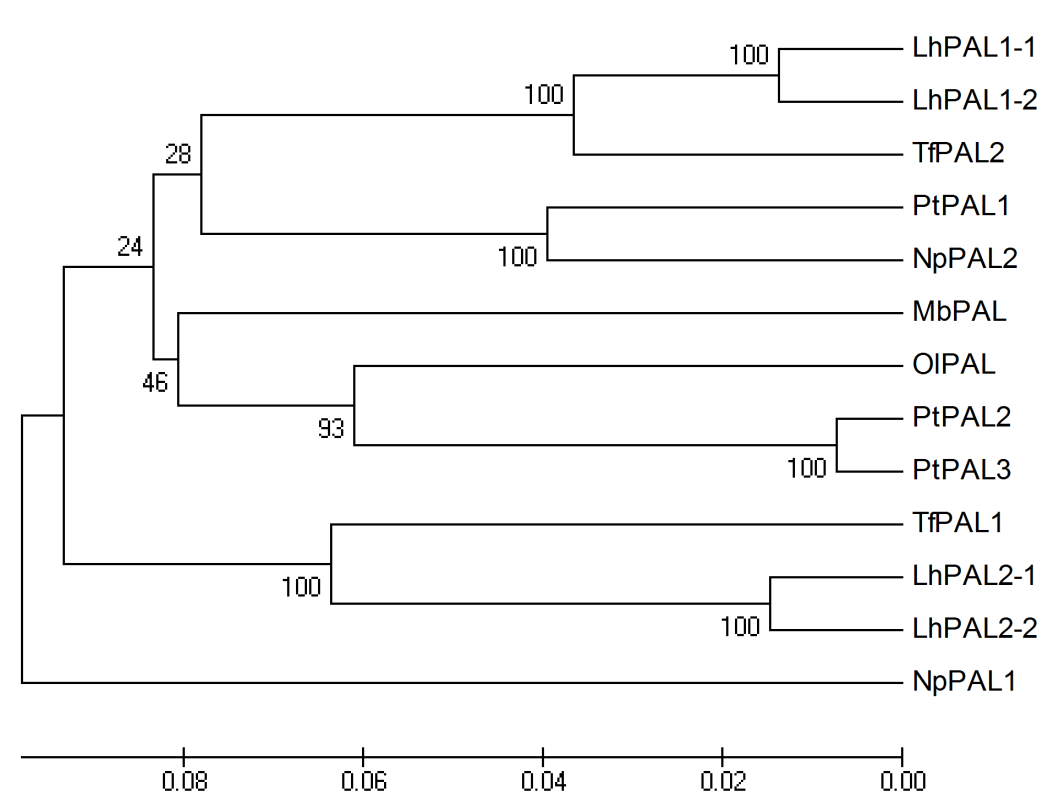


LhPAL1-1: *Lilium hybrid*, AB699153; LhPAL1-2: *L. hybrid,* AB699154; LhPAL2-1: *L. hybrid*, AB699156; LhPAL2-2: *L. hybrid*, AB699157; TfPAL1: *Tulipa fosteriana*, KF792730; TfPAL2: *T. fosteriana*, KP036454; NpPAL1: *Narcissus pseudonarcissus*, MF405173; NpPAL2: *N. pseudonarcissus*, MF405174; OlPAL: *Ornithogalum longebracteatum*, AHG06397; MbPAL: *Musa balbisiana*, BAG70992.
