# Supplementary material for: Transcriptome analysis of Polianthes tuberosa during floral scent formation
Source: PLoS One. 2018 Sep 5;13(9):e0199261. doi: 10.1371/journal.pone.0199261 (PMC6124719; doi:10.1371/journal.pone.0199261)
Supplement: Supplementary file 10 [file pone.0199261.s010.docx]

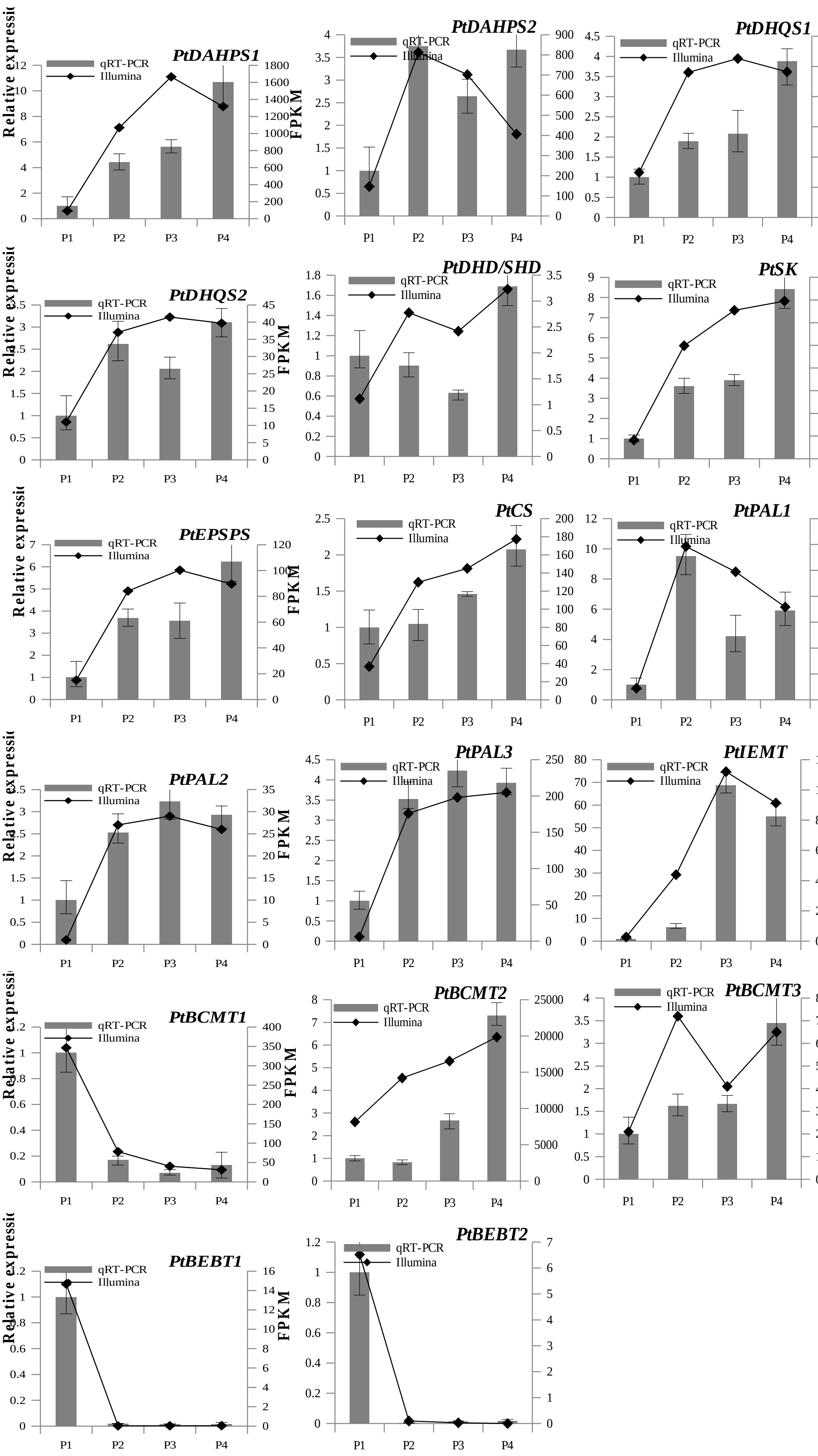
PtDAHPS1
PtDAHPS2
PtDHQS1
PtDHQS2
PtDHD/SHD
PtSK
PtEPSPS
PtCS
PtPAL1
PtPAL2
PtPAL3
PtIEMT
PtBCMT1
PtBCMT2
PtBCMT3
PtBEBT1
PtBEBT2
qRT-PCR
Illumina
Relative expressi
FPKM
P1
P2
P3
P4
