# Supplementary material for: Transcriptome analysis of Polianthes tuberosa during floral scent formation
Source: PLoS One. 2018 Sep 5;13(9):e0199261. doi: 10.1371/journal.pone.0199261 (PMC6124719; doi:10.1371/journal.pone.0199261)
Supplement: Supplementary file 11 [file pone.0199261.s012.docx]

```
PtTPS2   MAETFSKC...LAMSVGSSPVGIWNSGRRRGSK...............MAAYQRSSQAYYPQSISMIRQLKSVTP...VVREDYSAVKHT    69
PtTPS4   MAFYICSCSTTSFVPLKSVRSNRMLLCHAQGGARPKTSSIAAKTSPELTNNSERRSANYPPSVWEDGYIQSLR....IDFMGDDVDSQIR    86
PtTPS5   METQIQST.TASLLHISSPP......CENGGGD.................SVVRPSAGYHPSVWGDYFIIQQFPPSNIQEPEERINERVK    66
PtTPS6   MAFYICSCSTTSFVPLKSVRSNRMLLCHAQGGARPKTSSIAAKTSPELTNNSERRSANYPPSVWEDGYIQSLR....IDFMGDDVDSQIR    86

PtTPS2   ENIVKVKNILMQVDDPFESMLTVDYLQKLGIDYHFHEEIEAMLGSLHANVSGEQGQQVINDLFQVTLAFRLLRQAGYPISSEVLKKFTDN   159
PtTPS4   KLKEEVRHLLGEGTELVRLLQLVDTLQQLGVAYHFEKEINDVLTTIYRSKDNTI.MEMKDDLYATSLFFRLLREHGFHISPDIFNNFKDE   175
PtTPS5   ALVTQVKVLLKNCDTQLQEIQLITALQHLGVAYHFEKEIDEALSRIHNDHVD......SDDLHFVSLRFRLLRQHGYYVPCDIFRKFKDD   150
PtTPS6   KLKEEVRHLLGEGTELVRLLQVVDALQQLGVAYHFEKEIKDVLTTIYRSKDNTI.MEMKDDLYATSLFFRLLREHGFHISPDIFNNFKDE   175

PtTPS2   KGGFKSTLATDVRGLLGLYEASYLNT.GENILDEANEFSSKHLKSCIE..HLEPNVATLVRHTLMHPRHMTISRYNVRQYLDSCQTGHG.   245
PtTPS4   KGSFNTNLSNDIKGILSLYEASFLAIEGEDQMDEARQFTTDCLKDLTKRFLIEPSLKEKVSHALELPLHWRMQRLHTRWFIDQYEREED.   264
PtTPS5   EGNFKAIFNNDAKALLSLYEAAYLSIPGEDILDDAANFAKKHLQSMHN..NSETALAMQVSQALHLPLRRRMEILEARNYIDIYQEDEDN   238
PtTPS6   KGSFNTNLSNDIKGILSLYEASFLAIEGEDQMDEARQFTTDCLKDLTKRFLIEPSLKEKVSHALELPLHWRMQRLHTRWFIDQYEREED.   264

PtTPS2   ...VLEELARLDFNLVQSLHQKELRQISSWWESTGLADELKFARNQPLKWYLWSVTILEHSEFSKCRMEMTKVIAFIYIIDDIFDVYGRP   332
PtTPS4   MDPSLLKLAKLDFNMLQGIYIKELKELSRWWMNLGLGQSLGFARDRLVENYLWAVGWAFEPKFSRTREAMTKANCFITMIDDVYDVYGSQ   354
PtTPS5   RNDVVLELAKLDFHLLQLLHREEAKTITKWWKELGLASKLTFARDRVVECYFWTLGVYFEPHYAQARLILTKVISHFSLMDDIYDAYGTV   328
PtTPS6   MDPSLLKLAKLDFNMLQGIYIKELKELSRWWMNLGLGQSLGFARDRLVENYLWAVGWAFEPKFSRTREAMTKANCFITMIDDVYDVYGSQ   354
                                                                                      DDXXD

PtTPS2   EELELFTSAISKWDLSAVETLPAYMQACYTALYNITNEIAKTVSKEQGWNPVNSLKKPWIRLCKAFLIESRWLRFNHIPKADEYMKNAVI   422
PtTPS4   EELELFTDAVIRWEIGAIEQLPNYMKILFIALFNTTNDTAYKTLKLKGIDVLRYLKRAWKDLCKSYLIEAKWFAEGYTPTLKEYLDNAWI   444
PtTPS5   EELSIYTELIQRWNLDGVDQLNESLKLQYLALYDLVNDLENELANEHKSYRVRYLKEGIKSTCSAWFKESQWRDAGYVPSLDEYLSVTTV   418
PtTPS6   EELELFTDAVIRWEIGAIEQLPNYMKILFIALFNTTNDTAYKTLKLKGIDVLRYLKRAWTDLCKSYLIEAKWFAEGYTPTLKEYLDNAWI   444

PtTPS2   SSGVHVVLMHIFFLLGEGITKENVEFLESNPALLSCPATILRLWDDLGSAKDENQEGFDGSYVECYMNEYPQHSAQNAREHVKQIISGSW   512
PtTPS4   SISGHIALTHAYCTSDDLT.EEALECFNFYPDIVCHSSMLLRLHDDFATSTAELERGDVPKSIQCYMHENGVTEAI.AREQISEMVTSTW   532
PtTPS5   SATYPALACVSFVGMGEIATEQAFEWIHNFPRCVEAICIICRLLDDIVSHELEQERDHVASAIQCYMKEHGASSQE.ACKKSLEMIENAC   507
PtTPS6   SISGHIALTHAYCTSDDLT.EEALECFNFYPDIACHSSMLLRLHDDLATSTAELERGDVPKSIQCYMHENGVTEAI.ACEQISEMVTSTW   532

PtTPS2   KNLNKECFSRDHFASNLVTSSLNISRMTEVMYRYDDNQNLP..IMEDYINVLLFKSI                                 567
PtTPS4   KKLNGDRIANSSFVESFKKVALNIPRMSQCIYQNGDGYGEPGGKTKDQIFSLLIDSIP                                590
PtTPS5   KELNQECLTQKTISMHLLMRVVNLARVMETMYREYDTYTHSSTTLKDYITLLLVQPIM                                565
PtTPS6   KKLNGDRIANSCFVESFKKVALNIPRMSQCIYQNGDGYGEPGGKTKDQIFSLLIDSIP                                590
```
